# Engineering yeast for anticancer drug biosynthesis

**DOI:** 10.1038/s41421-026-00937-x

**Published:** 2026-11-09

**Authors:** Nicolas Gautron, Nicolas Papon, Vincent Courdavault

**Affiliations:** 1 Biomolécules et Biotechnologies Végétales, BBV, EA2106, Université de Tours, Tours, France; 2 Univ Angers, Univ Brest, IRF, SFR ICAT, Angers, France

**Keywords:** Plant molecular biology, Molecular biology

**In** **a** **recently published** ***Science* article,** **Shen et al. established a modular yeast platform for the de novo biosynthesis of a direct precursor of etoposide and teniposide from glucose. This advance provides unprecedented perspectives for a sustainable supply chain for these important anticancer drugs.**

For centuries, medicinal plants were used raw, as extracts, or macerated for their biological activities. Advances in chemistry and pharmacology have progressively enabled the isolation and administration of their active compounds, which remain the basis of modern medicine. Notable examples include paclitaxel, vinblastine, and podophyllotoxin, precursors of several anticancer drugs^1–3^. Access to these medicines is increasingly challenged by the low molecular yield and slow growth of plants or harvesting pressure. Developing more sustainable production methods is therefore critical to safeguarding their supply^4^. Producing these metabolites in cell factories engineered with heterologous genes could eliminate dependence on the original plant resource and enable stable, local production.

One major strategy is de novo synthesis, in which the target compound is produced in tailored yeasts from a simple carbon source^5^. However, unlike plants, *Saccharomyces cerevisiae* cannot necessarily reproduce the spatial and subcellular organization required for metabolite biosynthesis. As a result, metabolic intermediates can be diverted into side reactions, compromising final yields^6^. Extensive pathway engineering is therefore often required, as heterologous enzymes may show limited activity or expression in the new host^7^.

The production of etoposide and teniposide adds a further layer of complexity. While podophyllotoxin could in principle be produced de novo, several chemical steps are still required to obtain either drug^3^. A de novo platform therefore must combine the reconstruction of a long, branching biosynthetic pathway with downstream semisynthetic chemistry. In a report recently published in *Science*, Shen and colleagues hypothesized that reconstructing and optimizing the pathway toward the glycosylated intermediate 4′-demethyl-epipodophyllotoxin-4-*O*-glucoside (4′-DMEPI-4-*O*-Glc) could overcome limitations of plant-based production while reducing downstream chemistry to a single step^8^.

**Fig. 1:**
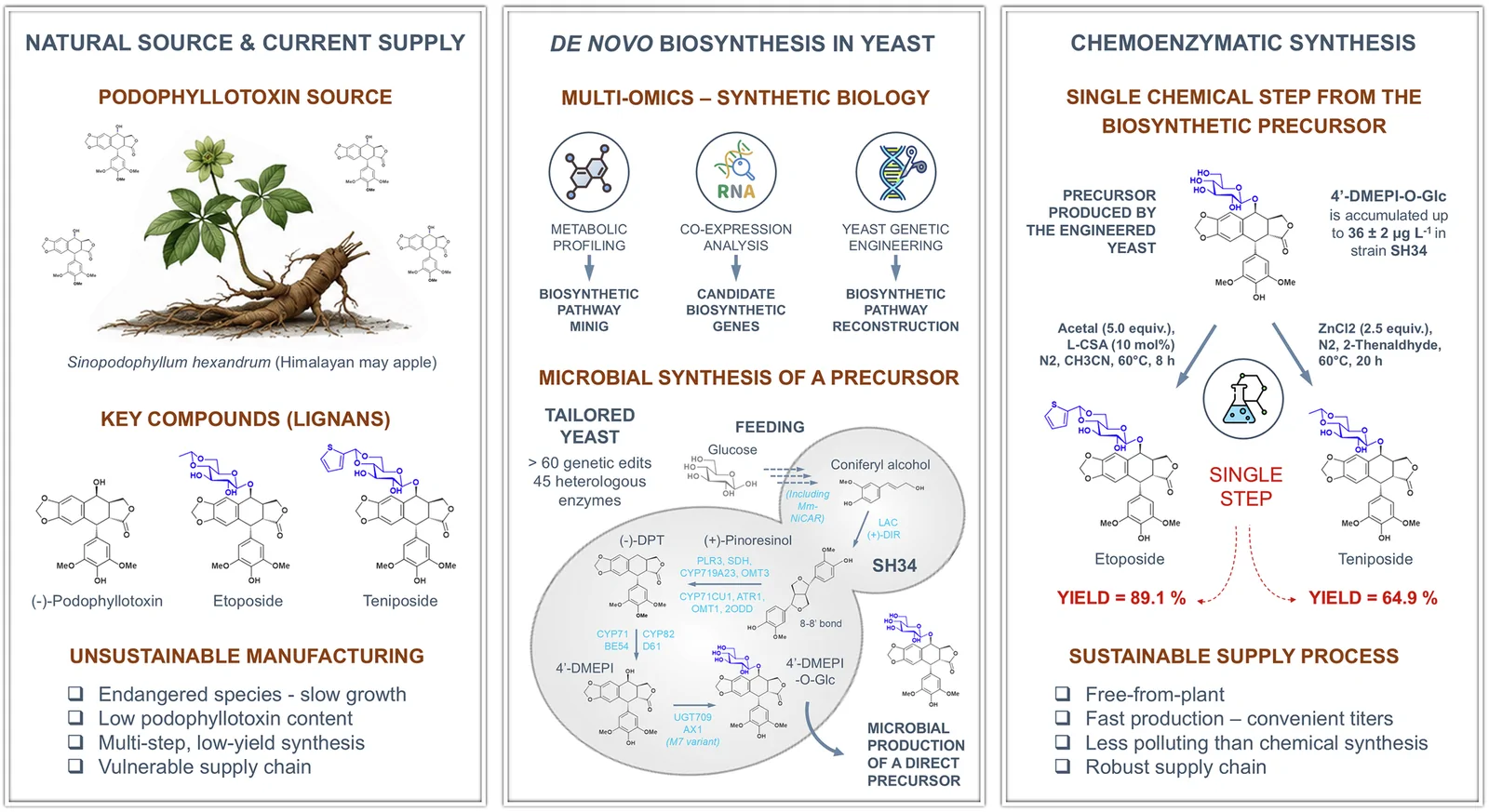
Towards a sustainable supply chain for production of the anticancer drugs etoposide and teniposide. Etoposide and teniposide are clinically important topoisomerase II inhibitors derived from the medicinal plant *Sinopodophyllum hexandrum*, whose production remains strongly dependent on plant-derived precursors. Here, Shen and colleagues combined plant genomics, enzyme discovery, structural biology, synthetic biology, and metabolic engineering to tailor a yeast strain capable of producing an advanced glycosylated precursor. The latter can be converted into either etoposide or teniposide through a single chemical step.

The authors first searched for the missing enzyme able to install this glucose unit. Screening 354 putative UDP-glycosyltransferase (UGT) genes from the *Sinopophyllum hexandrum* genome, they identified UGT709AX1, which catalyzes the regioselective C-4 glycosylation required to generate 4′-DMEPI-4-*O*-Glc (Fig. 1), closing a pathway gap remaining since the podophyllotoxin aglycone route was elucidated in 2015^3^.

A separate challenge was supplying the monolignol building block, coniferyl alcohol (CAL). Because the native plant 4CL/CCR/CAD route performed poorly in yeast, the authors engineered a non-natural pathway based on carboxylic acid reductases (CARs). Using an AlphaFold3-guided domain-swapping strategy with the peptidyl carrier protein (PCP)-domain repetition, they built eight functional chimeric CARs and selected *Mm-Ni*CAR. Increasing its gene copy number revealed caffeoyl aldehyde accumulation, which was resolved by screening ten aldehyde reductases and identifying ADH3 as the most effective. Further optimization of SAM and NADPH cofactor supply ultimately yielded 89 ± 2 mg/L CAL, a roughly 45-fold improvement over the initial plant-pathway strain.

The next milestone was pinoresinol (PIN), formed by oxidative 8-8′ coupling of two CAL units (Fig. 1). Because laccase-mediated coupling generates mixed linkage types, dirigent proteins (DIRs) are required to favor the stereospecific 8-8′ bond. Truncating both laccase and DIR sequences for improved yeast expression and increasing DIR dosage yielded 4 ± 0.1 mg/L of (±)-PIN, establishing a functional entry point into the aryltetralin lignan pathway.

Building the aryltetralin scaffold required screening 13 pinoresinol-lariciresinol reductase (PLR) orthologs and four secoisolariciresinol dehydrogenases (SDHs), which led to the identification of *Sh*PLR3 and *Sh*SDH as the most efficient enzymes. Two cytochrome P450s, CYP719A23 and CYP71CU1, were additional bottlenecks; N-terminal engineering and pairing with the *Arabidopsis thaliana* redox partner ATR1, combined with increased DIR copy number, raised production of (–)-deoxypodophyllotoxin (DPT) to 357 ± 58 µg/L.

With this DPT-producing chassis, the authors assembled four tailoring CYP450s and two UGTs into a combinatorial “plug-and-play” module, generating twelve podophyllotoxin-type compounds. Co-expression of CYP82D61, CYP71BE54, and UGT709AX1 yielded the direct precursor 4′-DMEPI-4-*O*-Glc in strain SH34 (Fig. 1). Because substantial aglycone remained unconverted, structure-guided engineering of the UGT709AX1 active site produced the M7 variant, increasing the precursor titer nearly 4-fold to 132 ± 8 µg/L.

The final step converts this glycosylated precursor into the active drugs. Instead of the multi-step protection-deprotection sequences typically required for glycosidic bond formation, the authors applied a single mild acetalization: acid-catalyzed reaction with acetaldehyde diethyl acetal delivered etoposide in 89.1% yield, while ZnCl₂-mediated reaction with 2-thiophenecarboxaldehyde delivered teniposide in 64.9% yield (Fig. 1).

Overall, this study does not yet solve the industrial supply problem for etoposide and teniposide: titers of the key glycoside precursor remain in the low hundreds of micrograms per liter, several orders of magnitude below fermentation-scale requirements. The authors themselves identify the remaining bottlenecks. The laccase–DIR coupling node is only partially stereoselective in yeast, suggesting that spatial co-localization of the two proteins, rather than DIR dosage alone, will be needed to enforce stereocontrol in a heterologous cytosol lacking the plant’s native architecture. Further downstream, competition between CYP71CU1 and deoxypodophyllotoxin synthase for a shared intermediate diverts flux away from the podophyllotoxin-type scaffold, while increasing CYP71BE54 or improved UGT709AX1-M7 dosage failed to raise precursor titers. This indicates that enzyme engineering alone has reached diminishing returns and that upstream flux partitioning is now limiting. Closing these gaps will likely require enzyme scaffolding or compartmentalization rather than further gene copy-number tuning, which typically requires substantial post-discovery process engineering before reaching commercially relevant titers^9,10^.

What this work does deliver is conceptually significant rather than merely incremental. It closes a ten-year gap since the etoposide aglycone pathway was first elucidated by identifying UGT709AX1, the glycosyltransferase used to install the exact glucose linkage required for etoposide and teniposide synthesis. It thereby relocates the most stereochemically demanding step — enzyme-mediated, *S*-configured C-4 glycosylation — from the chemist’s flask into microbial metabolism, reducing a downstream sequence that traditionally requires > 10 protection-deprotection steps and gets < 20% overall yield to a single, high-yielding chemical transformation. This chemoenzymatic division of labor, pairing a fully biosynthetic, stereochemically faithful precursor with a minimal final chemical step, offers a pragmatic template for essential-medicine supply chains compared with pursuing pure fermentation or synthesis alone. Because the platform’s oxidative tailoring and glycosylation modules are interchangeable, the same chassis can generate podophyllotoxin-type analogs not found in nature, enabling structure-activity exploration beyond plant extraction or classical synthesis. The study’s lasting contribution may therefore be less the specific titers reported than the modular framework itself, a reusable blueprint for bringing other geographically and ecologically constrained plant anticancer agents within reach of secure, scalable production.
